# ﻿The floral morphology of four Chinese *Bambusa* species (Poaceae, Bambusoideae) previously described only from vegetative material

**DOI:** 10.3897/phytokeys.213.95614

**Published:** 2022-11-16

**Authors:** Qiao-Mei Qin, Zhuo-Yu Cai, Jing-Bo Ni, Yi-Hua Tong, Nian-He Xia

**Affiliations:** 1 Guangdong Eco-engineering Polytechnic, Guangzhou 510520, China Guangdong Eco-engineering Polytechnic Guangzhou China; 2 Key Laboratory of Plant Resources Conservation and Sustainable Utilization, South China Botanical Garden, Chinese Academy of Sciences, Guangzhou 510650, China South China Botanical Garden, Chinese Academy of Sciences Guangzhou China; 3 University of Chinese Academy of Sciences, 100049, Beijing, China University of Chinese Academy of Sciences Beijing China

**Keywords:** Bambuseae, epitype, pseudospikelet, taxonomy

## Abstract

Due to their specialised flowering biology where frequent or even annual flowering is uncommon, reproductive materials of bamboos are not always available, so hampering taxonomic interpretation and research into other aspects. *Bambusacontracta*, *B.corniculata*, *B.cornigera* and *B.subtruncata* were established only based on vegetative materials and flowering or fruiting material has been hitherto unknown. The floral morphology of these four species is described for the first time and, correspondingly, epitypes are designated to support a more complete interpretation of the species.

## ﻿Introduction

The subfamily Bambusoideae (Poaceae) includes forest grasses that number more than 1680 species in 127 genera classified into three tribes, viz., Olyreae, Bambuseae and Arundinarieae (Sungkaew et al. 2009; Triplett and Clark 2010; Zhang et al. 2012; Vorontsova et al. 2016; Clark and de Oliveira 2018). *Bambusa* Schreb. is a genus of Bambuseae including more than 100 species and is distributed in tropical and subtropical Asia (Xia et al. 2006). In China, there are about 80 *Bambusa* species of high practical and economic value (Jia et al. 1996; Xia et al. 2006).

Phylogenetic studies have shown that *Bambusa* is not monophyletic and it has complicated genetic relationships with *Dendrocalamus* Nees and *Gigantochloa* Kurz ex. Munro (Goh et al. 2010, 2011, 2013; Guo 2010; Zeng 2014; Qin 2019). Therefore, the reticulate alliance amongst these three genera has been called the *Bambusa*-*Dendrocalamus*-*Gigantochloa* complex (or ‘BDG’ complex) (Goh et al. 2010, 2011, 2013). The vegetative morphology of *Bambusa* and its allies can sometimes be rather confusing. The main characters distinguishing these three genera are found in their floral morphology. *Bambusa* (except subgenus Dendrocalamopsis L.C. Chia & H.L. Fung) has elongate and disarticulating rachilla segments which in *Dendrocalamus*, *Gigantochloa*, as well as BambusasubgenusDendrocalamopsis, are absent (Wong 1995; Xia et al. 2006). In addition, *Gigantochloa* consistently features a firm filament tube while the other two typically have separate filaments (Wong 1995). Lack of detailed knowledge on the flowering characters, combined with the long flowering interval and frequent clump death after reproduction has resulted in floral material of a number of these bamboo species being unavailable. Thus, it is valuable to document the reproductive characteristics for every bamboo species, which benefits identification and the clarification of relationships amongst various taxa.

*Bambusacontracta* L.C. Chia & H.L. Fung, *B.corniculata* L.C. Chia & H.L. Fung, *B.cornigera* McClure and *B.subtruncata* L.C. Chia & H.L. Fung were published, based only on vegetative materials (McClure 1940; Chia and Fung 1981). The type of *B.cornigera* was collected from Wuzhou, Guangxi in 1928 (McClure 1940). The types of *B.subtruncata* (introduced from Xinyi, Guangdong), *B.contracta* and *B.corniculata* (introduced from Dongxing, Guangxi) were collected from cultivated material in the South China Botanical Garden (Chia and Fung 1981). Up to now, studies about *B.contracta*, *B.corniculata*, *B.cornigera* and *B.subtruncata* mainly focused on resource collection, protection, utilisation and evaluation of growth characteristics (Qiu et al. 2006; Wu 2008; Huang et al. 2013; Huang et al. 2014; Wu 2014; Huang et al. 2017), introduction and reproduction (Jin and Wang 1990; Zhang 2008), leaf epidermis micromorphology (Wang et al. 2002; Tao 2021), chromosome characteristics (Li et al. 2001; Jia et al. 2016) and vascular bundle morphology (Wen and Chou 1984; Fang et al. 1998). Studies on their floral morphology are unknown.

In this study, the pseudospikelet and floral morphology of these four *Bambusa* species are compared with those of closely-related species. Epitypes are designated here to support a more complete representation of the species.

## ﻿Materials and methods

All flowering materials were collected from plants cultivated in the Bambusetum of South China Botanical Garden, Chinese Academy of Sciences. They are deposited in the IBSC Herbarium of the South China Botanical Garden, as *Qin & Ni QQM 16* (*B.subtruncata*), *QQM 39* (*B.contracta*), *QQM 40* (*B.corniculata*) and *QQM 41* (*B.cornigera*). Dissection was carried out using a stereomicroscope (Olympus SZX16). Morphological comparisons were based on characters recorded in the relevant literature including protologues, as well as the study of type specimens. The specimens and photographic images were used for making descriptions.

## ﻿New epitypes and descriptions including flowering material

### ﻿ Bambusa contracta

Taxon classificationPlantaePoalesPoaceae

L.C. Chia & H.L. Fung (1981:376)

1DDD9810-7A54-5D90-B5ED-94CBF0BE432D

Figs 1 , 2

#### Holotype.

China, Guangdong Province: Guangzhou City, cultivated in South China Botanical Garden, Chinese Academy of Sciences (plants originally from Guangxi, Dongxing), 15 August 1978, *Nan-Zhu 2061* (IBSC!).

#### Epitype (designated here).

China, Guangdong Province: Guangzhou City, cultivated in South China Botanical Garden, Chinese Academy of Sciences, 31 March 2016, *Qin & Ni QQM 39* (IBSC!).

#### Description including flowering material.

Culms 5–6 m tall, 2–3 cm diameter, erect, apically drooping; internodes 34–57 cm long, plain green, initially slightly white waxy, with sparse long white hairs; wall ca. 3 mm thick; nodes flat, glabrous. Branch complement at mid-culm with a slightly dominant central branch and many subequal branches, those at culm base without thorny branchlets. Culm leaf sheath slightly white waxy, usually glabrous or basally dark brown hispid, apically arched with asymmetric sides; auricles unequal, oblong to lanceolate, undulate, wrinkled, larger auricle slightly slanted downwards, ca. 3 cm long, 0.7–1 cm wide, ca. 2 times larger than smaller one, bristles on the margin undulate; ligule ca. 2 mm high, margin sparsely dentate; blade erect, narrowly ovate, ca. 2/5 as long as sheath, base rounded, slightly overlapping with auricles for 2–3 mm, ca. 1/4 as wide as sheath apex, apex involute and acuminate. Foliage leaf sheath glabrous; auricles subovate, margin with long bristles; ligule very low, margin sparsely dentate; blade linear to linear-lanceolate, 10–15 cm long, 1.3–1.5 cm wide, abaxial surface densely pubescent, adaxial surface glabrous. Pseudospikelets fasciculate at each node of flowering branches, linear, sessile, basally subtended by several bud-bearing bracts, 2.5–3.5 cm long; florets 4–6, middle 2–4 florets fully developed; prophylls 1–2 mm long, 2-keeled, keels sparsely ciliolate; bracts 2–4, ovate to oblong, 3–5 mm long, glabrous, apically ciliolate, adaxial surface apex puberulent, obscurely 0–5-veined, apex obtuse to acute, mucronate or not; rachilla disarticulating between florets, segments compressed, 1.5–3 mm long, glabrous, lower segments distally inflated, upper segments distally only slightly inflated; glumes 1–3, oblong, 6–7 mm long, glabrous, sometimes adaxial surface apex puberulent, obscurely 11–13-veined, apex obtuse to acute, mucronate or not; lemma oblong, 7–10 mm long, glabrous, abaxial surface purple-spotted, 19–21-veined, apex acute mucronate, calluses no more than 5 mm long, glabrous; palea 9–11 mm long, 2-keeled, keels apically ciliolate, 4–5-veined between keels, each side 2-veined, apex truncate; lodicules 3, apex ciliate, anterior two broadly oblong, 2–3 mm long, posterior one narrowly oblong, 2–3 mm long; stamens 6, filaments filiform, anthers brown to yellowish, 5.5–7 mm long, apex retuse; ovary obovoid, 1.8–2 mm long, apex hispidulous, styles 3, 0.5–0.7 mm long, stigmas 3, plumose, 4–5 mm long.

**Figure 1.**
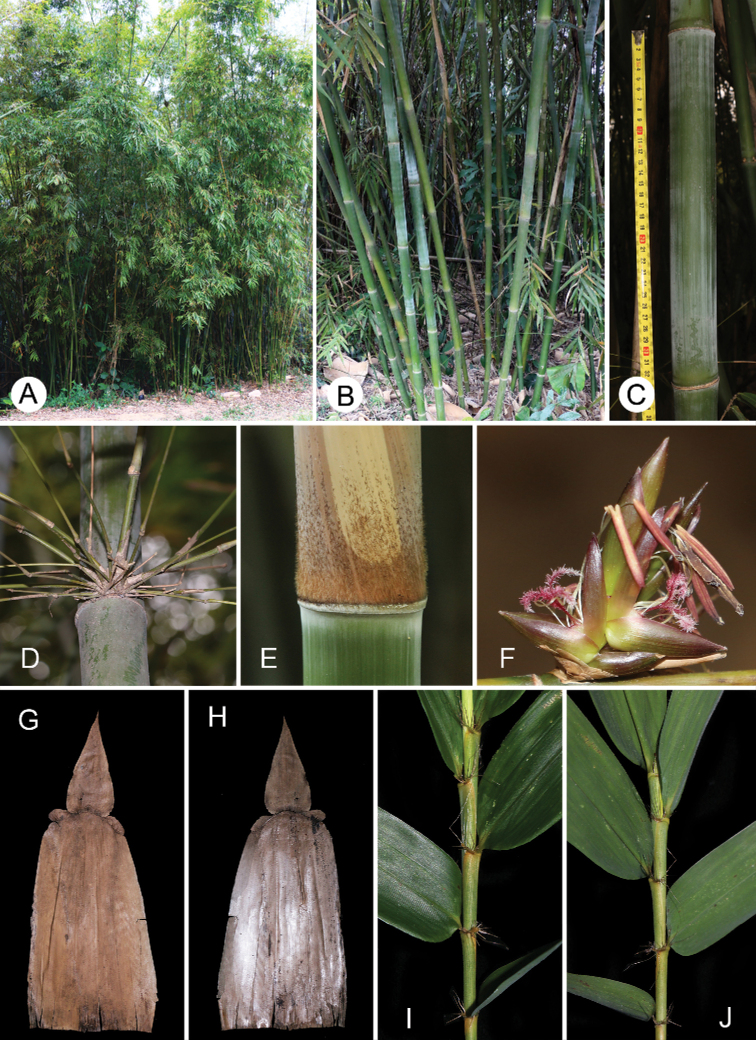
Vegetative morphology and pseudospikelets of *B.contracta***A** clumps **B** clump base **C** culm internode **D** branch complement **E** culm node with velvety hairs **F** pseudospikelets **G** culm leaf (abaxial view) **H** culm leaf (adaxial view) **I** the distal part of a leafy branch (upper side) **J** the distal part of a leafy branch (lower side).

**Figure 2.**
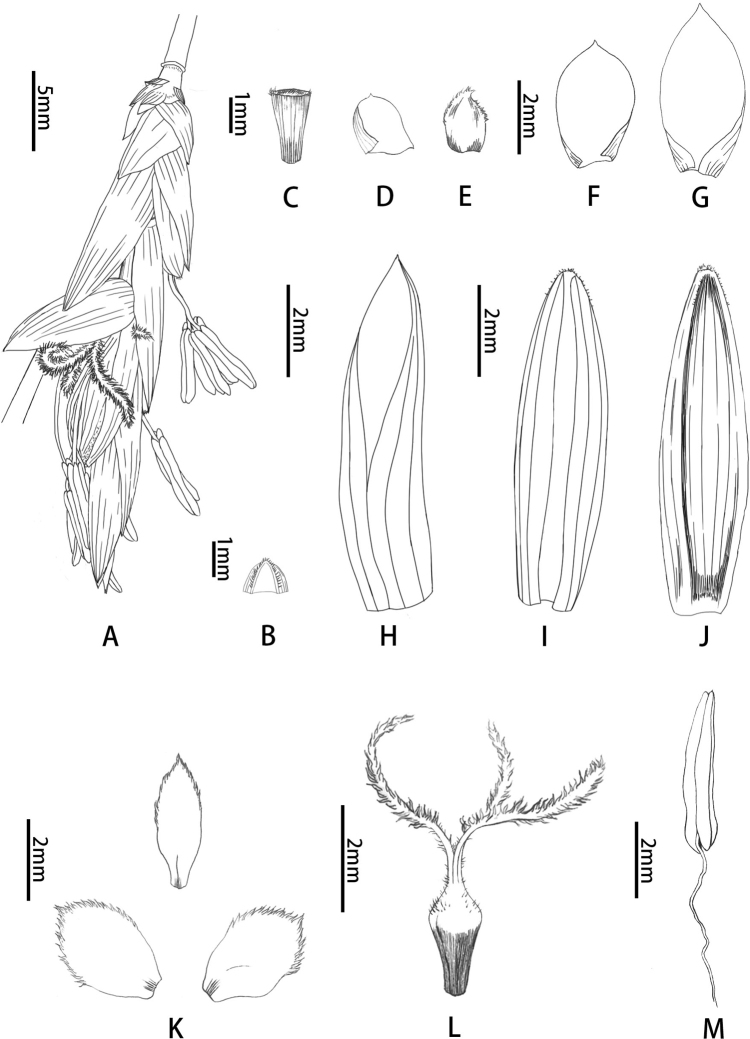
Floral morphology of *B.contracta***A** pseudospikelet **B** prophyll (abaxial view) **C** rachilla segment **D** bud-bearing bract (adaxial view) **E** axillary bud subtended by bract in (**D**) **F, G** glumes (adaxial view) **H** lemma showing margins **I** palea showing margins **J** back of palea **K** lodicules **L** pistil **M** stamen. Drawn by Qiao-Mei Qin.

#### Note.

We made a comparison between this species and its relative, *Bambusatextilis* McClure. The result shows that they share similar floral characters. *B.contracta* differs from *B.textilis* in having 4–5 veins between palea keels (versus 10), 2 veins on each wing of the palea (versus 4) and 11–13 veins on the glumes (versus 21).

### ﻿ Bambusa corniculata

Taxon classificationPlantaePoalesPoaceae

L.C. Chia & H.L. Fung (1981:368)

E163EC5C-EDA8-5DDB-AE28-DC93E2C155EF

Figs 3 , 4

#### Holotype.

China, Guangdong Province: Guangzhou City, cultivated in South China Botanical Garden, Chinese Academy of Sciences (plants originally from Guangxi, Dongxing), 15 August 1978, *Nan-Zhu 2599* (IBSC!).

#### Epitype (designated here).

China, Guangdong Province: Guangzhou City, cultivated in South China Botanical Garden, Chinese Academy of Sciences, 31 March 2016, *Qin & Ni QQM 40* (IBSC!).

#### Description including flowering material.

Culms ca. 8 m tall, 4–7 cm diameter, basally slightly zig-zag, apically drooping; internodes 20–32 cm long, basal ones conspicuously shorter, usually flat and shallowly grooved above branches, plain green, initially white waxy and with caducous sparse hispid hairs; wall ca. 8 mm thick; basal nodes with grey-white sericeous ring-like zones below and above sheath insertion, with short aerial roots. Primary branch bud horizontally elliptic, prophyll margins apically ciliate. Branch complement at lower culm nodes typically with only one branch bearing short, curved, weak, thorny branchlets; at mid-culm with 3 to several branches, central branch dominant. Culm leaf sheath glabrous, apex subtruncate, with a triangular protuberance on one shoulder; auricles unequal, lager auricle oblong or elliptic, ca. 8 mm wide, ca. 3 times larger than smaller one, margin with undulate bristles ca. 1 cm long; ligule ca. 3 mm high, short-fimbriate; blade erect, triangular or narrowly ovate, base 4/5 as wide as sheath apex. Foliage leaf sheath glabrous; auricles absent or tiny, semicircular to ovate, margin with undulate bristles; ligule very low, fimbriate; blade linear to lanceolate, 13–20 cm long, 1–2 cm wide, abaxial surface pubescent, adaxial surface glabrous. Pseudospikelets fasciculate at each node of flowering branches, linear, sessile, basally subtended by several bud-bearing bracts, 2–4 cm long; florets 7–9, middle 2–5 florets fully developed; prophylls ca. 4 mm long, 2-keeled, keels apically sparsely ciliolate; bracts 2–3, lanceolate, 4–6 mm long, glabrous, 1–9-veined, apex acute, mucronate or not; rachilla disarticulating between florets, segments compressed, 2–4 mm long, glabrous, apex ciliolate, lower segments distally inflated, upper segments distally only slightly inflated; glumes 1–3, ovate, ca. 7 mm long, glabrous, adaxial surface puberulent at the upper half, 15-veined, apex acute mucronate; lemma oblong, 8–12 mm long, glabrous, abaxial surface purple-spotted, 18–21-veined, apex acute mucronate, calluses ca. 0.5 mm long, glabrous; palea 8–13 mm long, 2-keeled, keels apically sparsely ciliolate, 4-veined between keels, each side 2-veined; lodicules 3, apex ciliate, anterior 2 obliquely oblong, 2.5–3 mm long, posterior one obovate, ca. 2.5 mm long; stamens 6, filaments filiform, anthers yellow to brownish, 5.5–6 mm long, apex retuse; ovary obovoid, 1.5–2 mm long, apex sparsely hispidulous, styles 3, 0.8–1 mm long, stigmas 3, plumose, 2.5–8 mm long.

**Figure 3.**
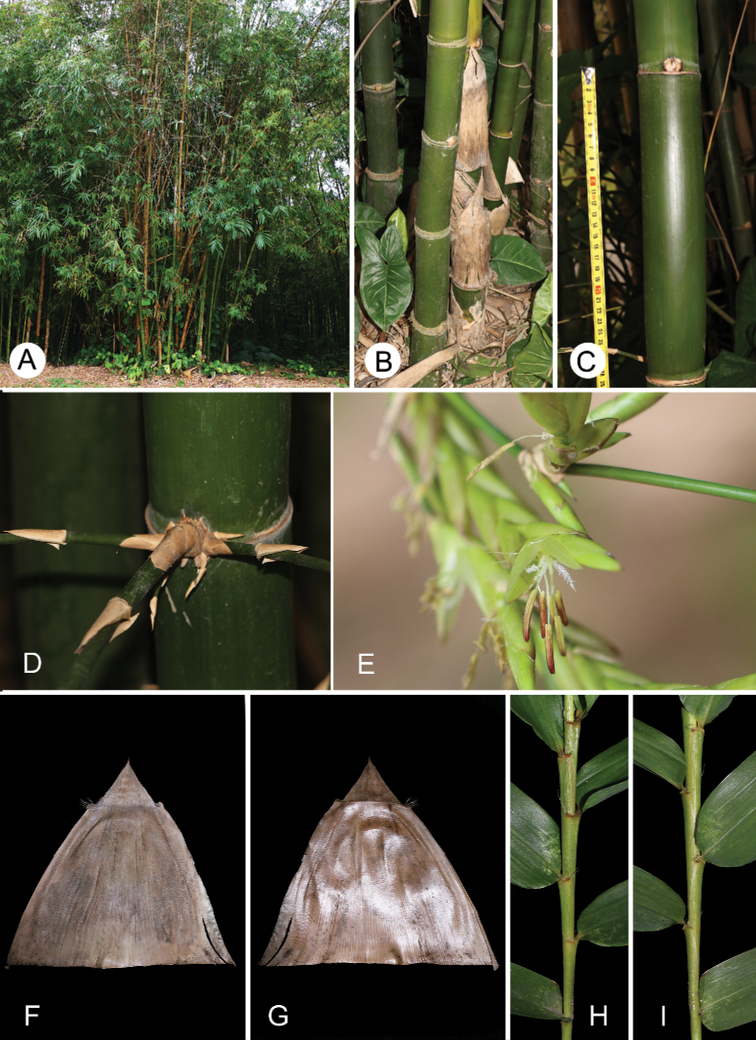
Vegetative morphology and pseudospikelets of *B.corniculata***A** clump **B** clump base **C** culm internode **D** thorny branches at culm base **E** pseudospikelets **F** culm leaf (abaxial view) **G** culm leaf (adaxial view) **H** the distal part of a leafy branch (upper side) **I** the distal part of a leafy branch (lower side).

**Figure 4.**
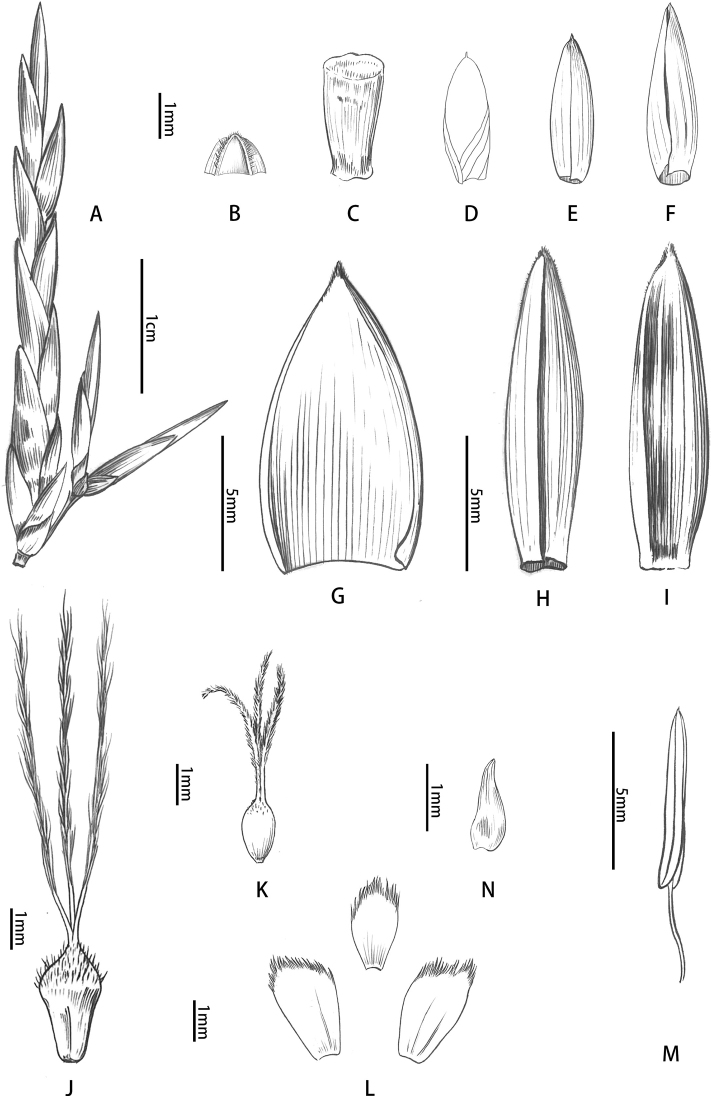
Floral morphology of *B.corniculata***A** pseudospikelets **B** prophyll (abaxial view) **C** rachilla segment **D** empty bract (adaxial view) **E, F** glumes (abaxial view) **G** lemma (adaxial view) **H** palea showing glabrous margins **I** back of palea **J** pistil **K** young pistil **L** lodicules **M** stamen **N** terminal floret. **D** drawn by Qiao-Mei Qin, **A–C** and **E–N** drawn by Ding-Han Cui.

#### Note.

This species is closely related to *Bambusagibba* McClure in vegetative morphology. However, *B.corniculata* can differ from *B.gibba* by apically acute (versus obtuse) bracts, glabrous (versus puberulent) rachilla segments, apically sparsely ciliolate (versus glabrous) keels of the palea, more veins on the palea and unstalked (versus stalked) ovary.

### ﻿ Bambusa cornigera

Taxon classificationPlantaePoalesPoaceae

McClure (1940:7)

7E7A92E8-F91F-567C-A88B-24BD6802EC16

Figs 5 , 6

#### Holotype.

China, Guangdong Province: Guangzhou City, cultivated in Lingnan University Bamboo Garden under BG 1833 (living type, originally from Guangxi, Wuzhou, Cangwu, Changzhou Island, West River, above Wuzhou), 10 September 1933, *H. Fung 20712* (US!).

#### Epitype (designated here).

China, Guangdong Province: Guangzhou City, cultivated in South China Botanical Garden, Chinese Academy of Sciences, 31 March 2016, *Qin & Ni QQM 41* (IBSC!).

#### Description including flowering material.

Culms 8–13 m tall, 6–8 cm diameter, basally straight or zig-zag, apically pendulous; internodes 24–28 cm long, slightly curved, basally somewhat swollen, glabrous, plain green, initially slightly white waxy; basal nodes with grey-white sericeous ring-like zones below and above sheath insertion. Branch complement at lower culm nodes with branchlets sometimes specialised into curved, weak thorns; at mid-culm with several branches, central 3 branches dominant. Culm leaf sheath ribbed-striate, with caducous, stiff, appressed, pale hairs above the middle, apex somewhat truncate; auricles equal, oblong, small, margins with fine bristles; ligule up to 3 mm high, entire, margin ciliate; blade erect, triangular to narrowly triangular. Foliage leaf sheath glabrous; auricles absent, without or with only a few bristles; ligule very low; blade lanceolate to oblong-lanceolate, 12–20 cm long, 2–3 cm wide, abaxial surface pubescent, adaxial surface glabrous. Pseudospikelets fasciculate at each node of flowering branches, linear, sessile, basally subtended by several bud-bearing bracts at base, 2.5–4.5 cm long; florets 10–12, middle 5–7 florets fully developed; prophylls 2–4 mm long, 2-keeled, keels apically sparsely ciliolate; bracts 2–3, lanceolate, 3–4 mm long, glabrous, apically ciliolate, sometimes adaxial surface apex puberulent, apex acute, mucronate or not; rachilla not disarticulating between florets, segments compressed, 2–3 mm long, glabrous, apex slightly ciliolate and flat; glumes 1–2, lanceolate to oblong, 5–7 mm long, glabrous, both surface apex puberulent, obscurely 11–13-veined, apex acute or obtuse, mucronate; lemma broadly elliptic, 10–12 mm long, glabrous, 19–23-veined, apex acute mucronate, calluses no more than 0.5 mm long, glabrous; palea 8–11 mm long, 2-keeled, keels apically ciliolate, 4–6-veined between keels, each side 2–3-veined; lodicules 3, apex ciliate, anterior 2 obliquely oblong, 3–3.5 mm long, posterior one narrower, ca. 3.5 mm long; stamens 6, filaments filiform, anthers yellow, 5–7 mm long, apex apiculate; ovary broadly ovoid, 1–2 mm long, apex hispidulous, style 1, 0.7–1 mm long, stigmas 3, plumose, 4–5 mm long.

**Figure 5.**
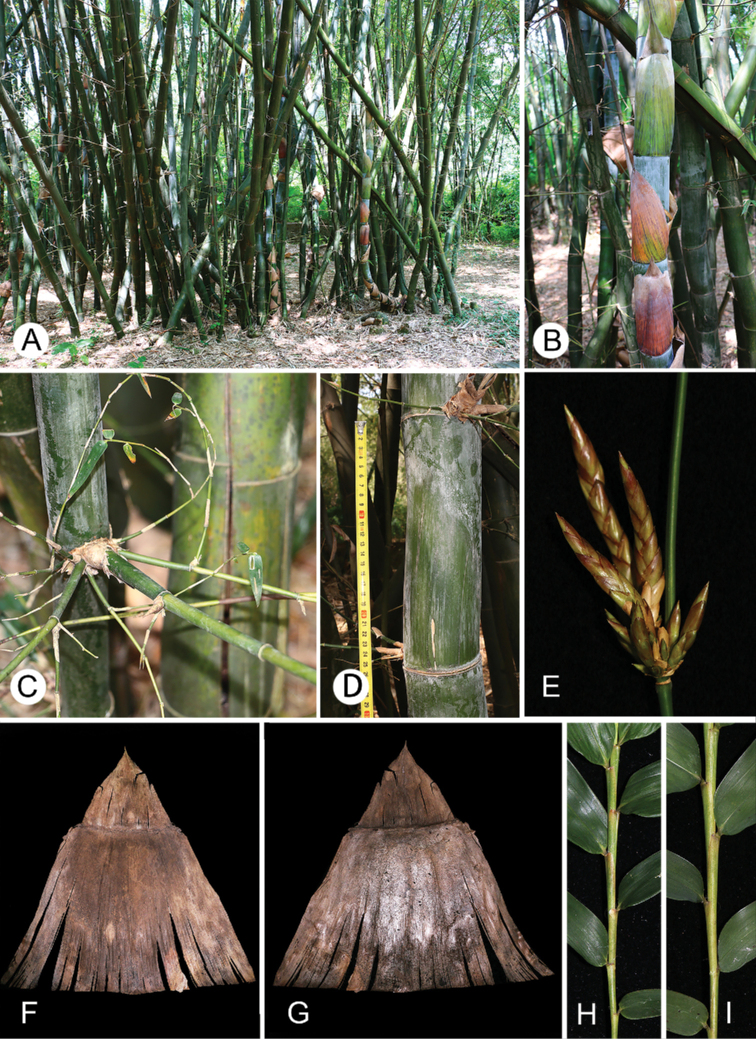
Vegetative morphology and pseudospikelets of *B.cornigera***A** clumps **B** young culm **C** thorny branches at culm base **D** culm internode **E** pseudospikelets **F** culm leaf (abaxial view) **G** culm leaf (adaxial view) **H** the distal part of a leafy branch (upper side) **I** the distal part of a leafy branch (lower side).

**Figure 6.**
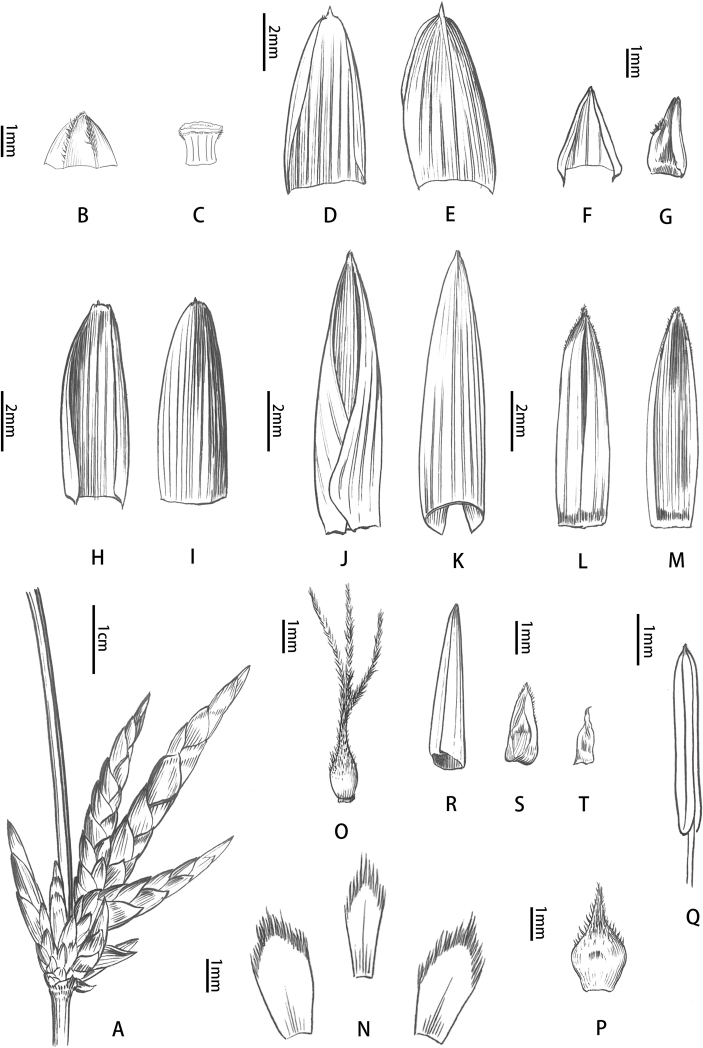
Floral morphology of *B.cornigera***A** pseudospikelets **B** prophyll (abaxial view) **C** rachilla segment **D** empty bract (adaxial view) **E** empty bract (abaxial view) **F** bud-bearing bract (adaxial view) **G** axillary bud subtended by bract in (**F**) **H** glume (adaxial view) **I** glume (abaxial view) **J** lemma showing margins **K** lemma (abaxial view) **L** palea showing margins **M** back of palea **N** lodicules **O** pistil **P** ovary **Q** stamen **R** lemma of subterminal floret **S** palea of subterminal floret **T** terminal floret. Drawn by Ding-Han Cui.

### ﻿ Bambusa subtruncata

Taxon classificationPlantaePoalesPoaceae

L.C. Chia & H.L. Fung (1981:378)

7AF091B0-6200-52C4-A471-3412833DA188

Figs 7 , 8

#### Holotype.

China, Guangdong Province: Guangzhou City, cultivated in South China Botanical Garden, Chinese Academy of Sciences (plants originally from Guangdong, Xinyi, Qingshui mountain), 5 August 1976, *Nan-Zhu 2312* (IBSC!).

#### Epitype (designated here).

China, Guangdong Province: Guangzhou City, cultivated in South China Botanical Garden, Chinese Academy of Sciences, 27 November 2015, *Qin & Ni QQM 16* (IBSC!).

#### Description including flowering material.

Culms 4–5 m tall, 2–2.5 cm diameter, basally nearly straight, apically slightly drooping; internodes 25–33 cm long, glabrous, initially slightly white waxy, green, basal ones typically with yellow stripes; wall ca. 7 mm thick; basal nodes with grey-white sericeous ring-like zones below and above sheath insertion. Primary branch bud broadly ovate, prophyll margins apically ciliate. Branch complement at mid-culm with many branches, central 3 branches dominant, those at culm base without thorny branchlets. Culm leaf sheath initially green with yellow stripes, glabrous or brown hispid especially near margins, apex subtruncate; auricles unequal, larger one about 2.5 times as large as smaller one, moderately to broadly elliptic, ca. 2 cm long, ca. 1.3 cm wide, wrinkled, margins with undulate bristles; ligule 1.5–2 mm high, margin ciliate; blade caducous, erect, triangular to narrowly triangular, base slightly rounded, overlapping with auricles for 6–7 mm, about 3/5 as wide as sheath apex. Foliage leaf sheath glabrous; auricles subovate or tiny, margin with undulate bristles; ligule low, entire; blade linear-lanceolate, 8–15 cm long, 0.9–1.3 cm wide, abaxial surface densely pubescent, adaxial surface glabrous. Pseudospikelets fasciculate at each node of flowering branches, linear, sessile, basally subtended by several bud-bearing bracts at base, 2.5–3 cm long; florets 9–10, middle 4–7 florets fully developed; prophylls ca. 3 mm long, 2-keeled, keels apically sparsely ciliolate; bracts 4–5, lanceolate, 3.5–10.5 mm long, glabrous, adaxial surface puberulent at the upper half, 5–15-veined, apex acute mucronate; rachilla disarticulating between florets, segments compressed, 4–5 mm long, apex ciliolate and flat; no glume; lemma oblong-lanceolate, 12–14 mm long, glabrous, 17–19-veined, apex acute mucronate, calluses ca. 1 mm long, glabrous; palea 12–13 mm long, 2-keeled, keels apically sparsely ciliolate, 4-veined between keels, each side 2-veined, apex slightly puberulent; lodicules 3, apex ciliate, anterior 2 obliquely oblong, 2.5–3.5 mm long, posterior one narrowly obovate, 2.5 mm long; stamens 6, filaments filiform, anthers brown to yellowish, 5–6.5 mm long, apex retuse; ovary obovoid, ca. 1.5 mm long, apex hispidulous, styles 3, 0.5–0.6 mm long, stigmas 3, rarely 4, plumose, 2.5–3 mm long.

**Figure 7.**
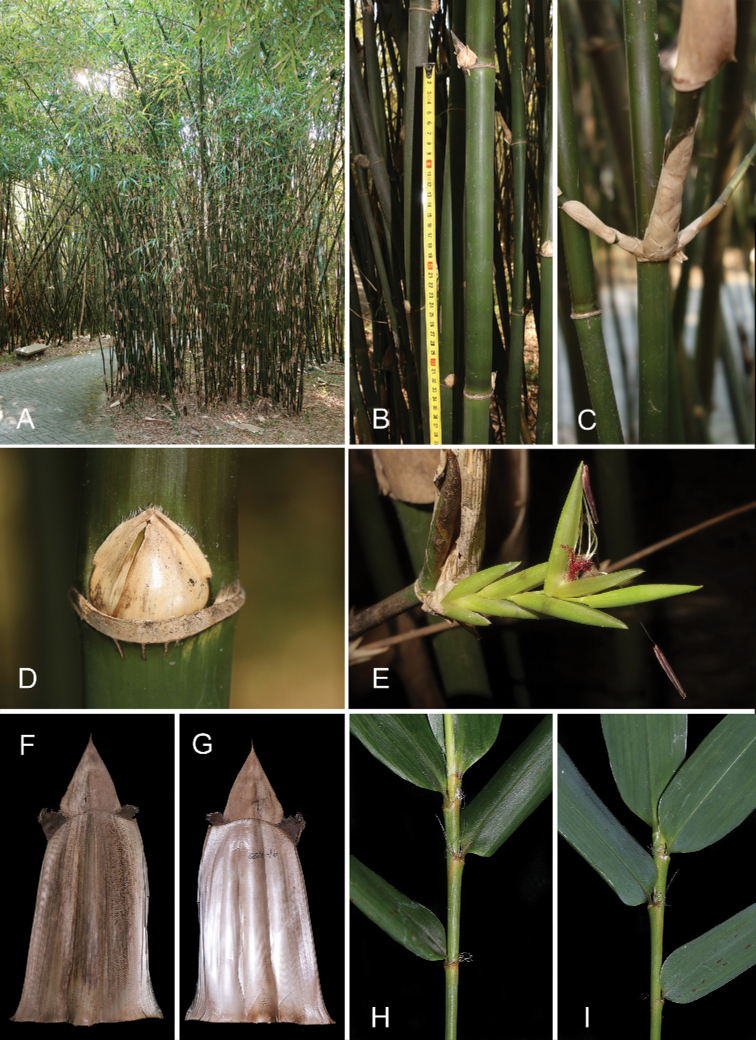
Vegetative morphology and pseudospikelets of *B.subtruncata***A** clump **B** culms internode **C** branch complement **D** primary branch bud **E** pseudospikelet **F** culm leaf (abaxial view) **G** culm leaf (adaxial view) **H** the distal part of a leafy branch (upper side) **I** the distal part of a leafy branch (lower side).

**Figure 8.**
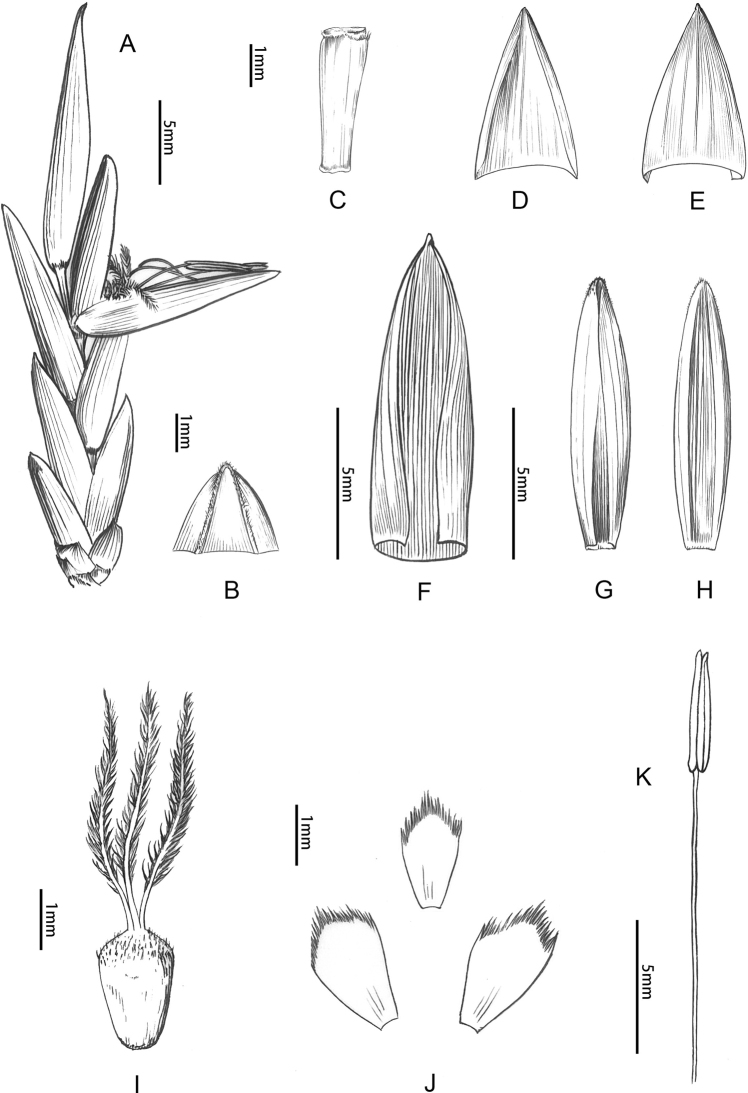
Floral morphology of *B.subtruncata***A** pseudospikelet **B** prophyll (abaxial view) **C** rachilla segment **D** empty bract (adaxial view) **E** empty bract (abaxial view) **F** lemma (adaxial view) **G** palea showing margins **H** back of palea **I** pistil **J** lodicules **K** stamen. Drawn by Ding-Han Cui.

#### Note.

After the comparison of floral characters between this species and a very similar congener, *Bambusatuldoides* Munro, the following differences were found: *B.subtruncata* has a flat rachilla segment apex (versus inflated), 4–5 bracts (versus 2), no glumes (versus usually just 1), an acute lemma apex (versus obtuse) and 2 veins on each side of the palea (versus 4 veins).

## Supplementary Material

XML Treatment for Bambusa contracta

XML Treatment for Bambusa corniculata

XML Treatment for Bambusa cornigera

XML Treatment for Bambusa subtruncata

## References

[B1] Chia LC Fung HL (1981) New species of the genus *Bambusa* Schreber from China. Acta Phytotaxonomica Sinica 19(3): 367–378.

[B2] Clark LG de Oliveira RP (2018) Diversity and evolution of the new world bamboos (Poaceae: Bambusoideae: Bambuseae, Olyreae). Proceedings of 11^th^ World Bamboo Congress. World Bamboo Organization, Xalapa, Mexico, 35–47.

[B3] Fang W Huang JQ Lu M Qian LY Fu WN (1998) Comparative anatomy on seventeen species of tufted bamboos. Journal of Zhejiang Forestry College 15(3): 225–231.

[B4] Goh WL Chandran S Lin RS Xia NH Wong KM (2010) Phylogenetic relationships among Southeast Asian climbing bamboos (Poaceae: Bambusoideae) and the *Bambusa* complex. Biochemical Systematics and Ecology 38(4): 764–773. 10.1016/j.bse.2010.07.006

[B5] Goh WL Chandran S Kamiya K Wong KM (2011) A natural hybrid between *Dendrocalamuspendulus* and *Gigantochloascortechinii* (Poaceae: Bambusoideae: Bambuseae) in Peninsular Malaysia. Gardens’ Bulletin (Singapore) 62(2): 223–228.

[B6] Goh WL Chandran S Franklin D Isagi Y Koshy KC Sungkaew S Yang HQ Xia NH Wong KM (2013) Multi-gene region phylogenetic analyses suggest reticulate evolution and a clade of Australian origin among paleotropical woody bamboos (Poaceae: Bambusoideae: Bambuseae). Plant Systematics and Evolution 299(1): 239–257. 10.1007/s00606-012-0718-1

[B7] Guo YB (2010) Taxonomic revision of the genus *Dendrocalamus* Nees (Poaceae: Bambusoideae) from China. PhD Thesis, Graduate School of the Chinese Academy of Sciences, China.

[B8] Huang CQ Huang T Liu W Chen L Peng J Teng SY Wang H (2013) The determination of frost resistance of 20 kinds of ornamental cluster bamboo. Journal of Hunan City University 22(2): 59–62. [Natural Science]

[B9] Huang DY Ling QM Xu ZG (2014) Bamboo resources and its utilization in China-Vietnam border area of Guangxi. World Bamboo and Rattan 12(3): 29–32.

[B10] Huang DY Huang DZ Li LJ Xu ZG Li J (2017) A study of bamboo species endemic to Guangxi and their protection. World Bamboo and Rattan 15(4): 13–17.

[B11] Jia LZ Feng XL Dai QH (1996) *Bambusa* Retz. corr. Schreber. In: Geng BJ Wang ZP (Eds) Flora Reipublicae Popularis Sinicae (Vol.9). Science Press, Beijing, 52–130.

[B12] Jia FX Zhou MB Chen R Yang HY Gao PJ Xu CM (2016) Karyotype and genome size in four bamboo species. Linye Kexue 52(9): 57–66.

[B13] Jin C Wang YY (1990) Introduction and productivity of clump bamboos. Journal of Bamboo Research 9(1): 43–54.

[B14] Li XL Lin RS Fung HL Qi ZX Song WQ Chen RY (2001) Chromosome numbers of some caespitose bamboos native in or introduced to China. Acta Phytotaxonomica Sinica 39(5): 433–442.

[B15] McClure FA (1940) New genera and species of Bambusaceae from Eastern Asia. Lingnan University Science Bulletin 9: 1–67.

[B16] Qin QM (2019) Taxonomic studies of *Sasa* Makino & Shibata and *Gigantochloa* Kurz ex Munro (Gramineae: Bambusoideae) from China. PhD Thesis, University of Chinese Academy of Sciences, China.

[B17] Qiu EF Peng ZH Wang C Zhou YG Ye GF Zheng R Liu FJ (2006) Evaluation on ecoadaptation of bamboo in urban greening. Acta Ecologica Sinica 26(9): 2896–2904.

[B18] Sungkaew S Stapleton CMA Salamin N Hodkinson TR (2009) Non-monophyly of the woody bamboos (Bambuseae; Poaceae): A multi-gene region phylogenetic analysis of Bambusoideae s.s. Journal of Plant Research 122(1): 95–108. 10.1007/s10265-008-0192-6 19018609

[B19] Tao XY (2021) Classification significance of phytolith morphology and microelement composition in bamboo leaves. MSc Thesis, Guilin University of Technology, China.

[B20] Triplett JK Clark LG (2010) Phylogeny of the temperate bamboos (Poaceae: Bambusoideae: Bambuseae) with an emphasis on Arundinaria and allies. Systematic Botany 35(1): 102–120. 10.1600/036364410790862678

[B21] Vorontsova MS Clark LG Dransfield J Govaerts R Baker WJ (2016) World checklist of bamboos and rattans. International Network of Bamboo and Rattan, Beijing, & the Board of Trustees of the Royal Botanic Gardens, Kew.

[B22] Wang RH Xia NH Lin RS (2002) Micromorphological study on leaf epidermis of *Bambusa* and *Dendrocalamus* (Poaceae: Bambusoideae). Journal of Tropical and Subtropical Botany 10(1): 22–26.

[B23] Wen TH Chou WW (1984) A report on the anatomy of the vascular bundle of bamboos from China (I). Journal of Bamboo Research 3(1): 1–21.

[B24] Wong KM (1995) The bamboos of Peninsular Malaysia. Forest Research Institute Malaysia, Kuala Lumpur.

[B25] Wu JL (2008) Collection of sympodial bamboos and evaluation of their cold resistance in Dahu Bamboo Garden. Journal of Bamboo Research 27(1): 19–26.

[B26] Wu WX (2014) The study on species and floristic characteristics of wetland plant in Guangxi, China. MSc Thesis, Guangxi Normal University, China.

[B27] Xia NH Jia LZ Li DZ Stapleton CMA (2006) 1. *Bambusa* Schreber. In: Wu ZY Raven PH (Eds) Flora of China (Vol.22). Science Press & Missouri Botanical Garden Press, Beijing & Saint Louis, 9–38.

[B28] Zeng Y (2014) Taxonomic studies of *Gigantochloa* Kurz ex Munro (Gramineae: Bambusoideae) from China. MSc Thesis, University of Chinese Academy of Sciences, China.

[B29] Zhang W (2008) Research on selection and in-vitro rapid propagation of cold-resistant sympodial bamboos. MSc Thesis, Chinese Academy of Forestry, China.

[B30] Zhang YX Zeng CX Li DZ (2012) Complex evolution in Arundinarieae (Poaceae: Bambusoideae): incongruence between plastid and nuclear GBSSI gene phylogenies. Molecular Phylogenetics and Evolution 63(3): 777–797. 10.1016/j.ympev.2012.02.023 22415014

